# Valine metabolite, 3-hydroxyisobutyrate, promotes lipid metabolism and cell proliferation in porcine mammary gland epithelial cells

**DOI:** 10.3389/fnut.2024.1524738

**Published:** 2025-01-10

**Authors:** Long Che, Le Liu, Mengmeng Xu, Zongze Fan, Lizhu Niu, Yujie Chen, Xueyuan Chang, Pan Zhou, Mengyun Li, Hongyu Deng, Wen Chen

**Affiliations:** ^1^College of Animal Science and Technology, Henan Agricultural University, Zhengzhou, China; ^2^College of Animal Science and Technology, Henan University of Animal Husbandry and Economy, Zhengzhou, Henan, China; ^3^Henan Swine Biobreeding Research Institute, Zhengzhou, Henan, China; ^4^School of Life Science and Engineering, Southwest University of Science and Technology, Mianyang, China

**Keywords:** valine, 3-hydroxyisobutyrate, mammary gland epithelial cells, lipid metabolism, sow

## Abstract

Improving mammary gland epithelial cells proliferation through nutrition is an important approach for enhancing sow milk production and piglet growth. An intermediate metabolite of valine, 3-hydroxyisobutyrate (3-HIB), regulates cellular lipid metabolism. In the present study, we investigated the effects of 3-HIB on porcine mammary gland epithelial cells proliferation and lipid metabolism. The addition of an appropriate concentration of 3-HIB significantly increased mammary gland epithelial cell proliferation and the expression of proteins associated with cell proliferation. Compared to the control group, the addition of 0.4–0.8 mM 3-HIB increased the expression levels of mTOR signaling pathway-related proteins and the cell cycle protein, Cyclin D1, while inhibiting the expression of the cell cycle arrest protein, P27. The addition of 0.8 mM 3-HIB increased the triglyceride and lipid droplet content in the cells. The addition of 3-HIB increased the expression of proteins related to *de novo* fatty acid synthesis and transport, resulting in a marked increase in most polyunsaturated fatty acids in the 3-HIB-added group. Compared to the control group, the addition of 0.8 mM 3-HIB increased the expression levels of the fatty acid oxidation-related proteins, ACSL and CAD, ultimately increasing cellular ATP synthesis. In summary, the addition of 0.8 mM 3-HIB to porcine mammary gland epithelial cells promotes cell proliferation by enhancing lipid metabolism and the expression of cell proliferation-related proteins.

## Introduction

Enhancing the lactation performance of sows is the key to improving the performance of weaned piglets and shortening the marketing age of growing-finishing pigs (1). The lactation ability of sows depends mainly on the number of porcine mammary gland epithelial cells (PMECs) and their secretory activity (2). Therefore, insufficient proliferation of PMECs, which leads to reduced milk production, is a major factor limiting the growth performance of piglets (2). Enhancing PMECs proliferation to improve lactation performance is imperative in pork production. During late pregnancy and the lactation period, PMECs begin to proliferate and differentiate into lobules and ducts, which form the mammary gland tissue (3). As lactation progresses, PMECs continue to divide and proliferate, producing more milk (4). Therefore, enhancing the proliferation of PMECs is the key to increasing milk production and improving the growth performance of piglets.

Valine is considered the second or third most limiting amino acid with the highest absolute absorption in the mammary gland tissue of sows during lactation (5). Only 60% of the ingested valine is exported in the form of milk, whereas up to 40% is involved in the internal physiological metabolism of the mammary gland (6). Thus, valine plays a key role in the physiological regulation of lactation in sows (2). Previous studies by our research group have found that increasing the level of valine in the diet of sows can increase the alveolar lumen area of the mammary gland tissue and milk production, thereby improving the survival rate and growth performance of nursing piglets (7). However, the underlying molecular mechanisms remain unclear and require further research.

The promotion of PMECs proliferation and an increase in the alveolar lumen area in sows by valine may be related to its metabolites. Using porcine intestinal epithelial cells as the research subject, the impact of valine supplementation on cellular lipid metabolism was investigated. Valine supplementation strongly promoted cellular fatty acid transport, thereby increasing triglyceride synthesis and cell proliferation (8). This study provides an important methodological reference for improving the composition of sow milk by nutritional means.

The entire valine metabolic process requires the participation of more than 10 enzymes (9). During this process, 3-hydroxyisobutyryl-CoA is formed and further converted into 3-hydroxyisobutyrate (3-HIB) under the catalytic action of 3-hydroxyisobutyrate-CoA deacylase (HIBCH) (9). However, silencing the expression of the *HIBCH* gene using siRNA to reduce 3-HIB synthesis leads to a substantial decrease in cellular triglyceride synthesis (8). This indicates that 3-HIB may be a key metabolite mediating the regulation of cellular lipid metabolism via valine. Recent studies have shown that when valine metabolites are oxidized in the mitochondria, almost all metabolic products enter the tricarboxylic acid cycle for oxidative energy supply (10). However, 3-HIB is the only unique substance that can “escape” mitochondrial oxidation and participate in the regulation of cellular lipid metabolism, playing an important role in the regulation of lipid synthesis (11). Currently, 3-HIB has been confirmed to regulate fatty acid transport and promotes cell proliferation in human and mouse skeletal muscle cells (12, 13), as well as in porcine intestinal epithelial cells (8). The proliferation of PMECs is fundamental for mammary tissue development to ensure milk production. In PMECs, lipid metabolism, especially fatty acid *β*-oxidation in cells, is an important source of energy required for cell proliferation (14). Therefore, it was hypothesized that 3-HIB could regulate cell proliferation by improving lipid metabolism in PMECs. This research elucidates the mechanism by which valine regulates the proliferation and lipid metabolism of PMECs, but also provides a reference for the future application of 3-HIB in sow production.

## Materials and methods

### Chemicals and reagents

Cell culture-related reagents, including Dulbecco’s modified Eagle’s F12 Ham medium (DMEM/F12), fetal bovine serum (FBS), antibiotics, trypsin/EDTA, and sterile phosphate-buffered saline (PBS), were procured from Invitrogen (Carlsbad, CA). The 3-HIB was obtained from Sigma-Aldrich (St. Louis, MO). Insulin, hydrocortisone, epidermal growth factor, oil Red O staining kit, Cell Counting Kit-8 (CCK8), cell lysis buffer, BCA protein assay kit, Hoechst 33342, and EdU enhancement detection kit were purchased from Beyotime Biotechnology (Shanghai, China). Triglyceride kits and Western blotting reagents, including transfer and electrophoresis buffers, and blocking solution, were purchased from Applygen Technologies, Inc. (Beijing, China). Anti-cyclin D1, anti-caspase 3, anti-P27, anti-CD36, and anti-ACC antibodies were purchased from Cell Signaling Technology (Beverly, MA). Anti-FASN, anti-SLC27A1, anti-FABP3, anti-LPL, and anti-DGAT were purchased from Abcam (Cambridge, UK). Anti-CPT1, anti-ACSL, and anti-CAD antibodies were purchased from Proteintech (Proteintech Group, United States). Anti-rabbit IgG, anti-mouse IgG, and anti-*β*-actin antibodies were purchased from AmyJet Scientific (Wuhan, China).

### Cell culture and treatment

The PMECs were conducted exactly as previously described (15). The method for culturing PMECs was based on a previous study (16). The culture medium contained 10% FBS, 5 μg/mL insulin, 1 μg/mL hydrocortisone, 5 ng/mL epidermal growth factor (EGF), 1% penicillin and streptomycin and cells were maintained at 37°C in a humidified atmosphere with 5% CO_2_. The cell culture medium was replaced every 48 h, and the cells were passaged for further culture using 0.25% trypsin–EDTA when they reached 90% confluence. Referring to our previous research (16), PMECs were reseeded at 6.0 × 10^3^ cells/well in 96-well plates or 2.5 × 10^5^ cells/well in six-well plates. The 3-HIB was dissolved into a stock solution using PBS, which was then diluted according to the specific concentrations used in the experiment.

### Cell viability assay

The PMECs were cultured in a 96-well culture plate and treated with different concentrations of 3-HIB (0, 0.2, 0.4, 0.8, and 1.6 mM) for 4, 8, 16, 24, and 48 h, after which 20 μL CCK-8 reagent was added to each well and incubation was continued for a further 2 h. The absorbance at 450 nm in each well was measured using an enzyme-labeling instrument (Thermo Fisher Scientific, Carlsbad, CA). Cell proliferation was detected using EdU Cell Proliferation Detection Kit according to the manufacturer’s protocol. Briefly, after treating the cells with 3-HIB for 16 h, 10 μM EdU reagent was added to each well and incubation was continued for 2 h. The culture medium was the discarded and the cells were fixed with 4% paraformaldehyde for 15 min. The fixative was removed and the cells were washed with PBS and treated with 0.3% Triton X-100 for 10 min. After discarding the Triton X-100 and washing twice, 100 μL of Click reaction solution from EdU Cell Proliferation Detection Kit was added to each well and incubated for 30 min. Click wash buffer was used to wash the cells three times. Subsequently, cell nuclei were stained with Hoechst 33342 for 10 min. After nuclear staining, the cells were washed three times and observed under a fluorescence microscope (NIS-Elements, Nikon, Japan).

### Oil red O staining

Cells were treated with different concentrations of 3-HIB (0, 0.2, 0.4, 0.8, and 1.6 mM) for 72 h and fixed with 4% paraformaldehyde for 30 min. The cells were washed with isopropanol and stained with Oil Red O staining solution for 30 min, followed by two washes with isopropanol and ultrapure water, respectively. The cell nuclei were stained with hematoxylin for 10 min and rinsed. Finally, the cells were examined and photographed under a microscope (NIS-Elements, Nikon, Japan).

### Analysis of fatty acid composition

The PMECs were cultured in six-well culture plates and treated with concentrations of 3-HIB concentration (0 mM or 0.8 mM) for 12 h. After washing with PBS, cells were collected using a cell scraper. The number of cells in each replicate was recorded to correct for fatty acid composition analysis. Determination of fatty acid composition in the cells was performed according to methods used in previous studies (8). The method for determining fatty acid composition included steps such as extraction of fatty acids from cells, fatty acid methylation, and gas chromatography. The results were calculated based on the corresponding peak area ratios.

### Western blot analysis

The cells were seeded in six-well plates and treated with different concentrations of 3-HIB (0, 0.2, 0.4, 0.8, and 1.6 mM) for 48 h. After treatment, cell samples were collected using a cell lysis buffer. The collected cell samples were centrifuged at 12,000 × *g* for 15 min at 4°C and the supernatant was used for concentration determination and target protein expression analysis. A BCA protein concentration assay kit was used to determine the cellular protein concentration following the manufacturer’s instructions. Protein expression levels were determined based on previous studies (17); methods included sample preparation, protein electrophoresis, transfer, blocking, and antibody incubation. Immunoreactivity was visualized with chemiluminescent HRP substrate (Millipore, Billerica, MA) using the VersaDoc imaging system (Tanon, China). The protein loading amount was 20 μg per well, with electrophoresis parameters set at a low voltage of 70 V for 30 min, followed by a high voltage of 110 V for 1 h. A constant-current transfer membrane was used at 250 mA for 90 min. A rapid blocking solution (Beyotime Biotechnology) was used for 30 min. The primary antibody was incubated at 4°C for approximately 16 h, followed by incubation with the secondary antibody at 25°C for 1 h. Band intensities were obtained using the ImageJ software after normalization to *β*-actin.

### Statistical analysis

All data are presented in the Tables and Figures as means ± standard error of the mean (SEM). The SPSS statistical software program (v. 19.0, SPSS; IBM SPSS Company, Chicago, IL, United States) was used to test the significance of the data using one-way analysis of variance, and Tukey’s test was used to determine the differences among the groups. Statistical significance was set at *p* < 0.05.

## Results

### Effect of 3-HIB supplementation on cell proliferation

**Figure 1:**
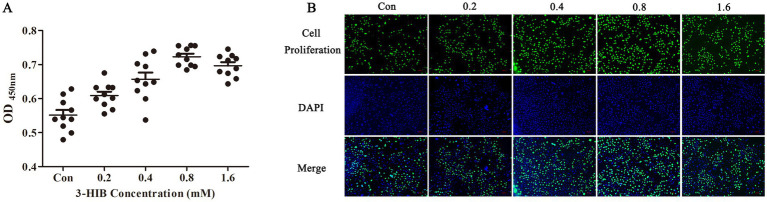
Effect of different 3-HIB concentrations on cell proliferation after 16 h. **(A)** Detection of cell proliferation using the CCK-8 assay. **(B)** Detection of cell proliferation using the EdU assay.

The effects of different concentrations of 3-HIB on cell proliferation were tested after 2, 4, 8, 16, 24, and 48 h (Table 1). The 3-HIB treatment did not affect cell proliferation during the first 8 h (*p* > 0.05). When treated with 3-HIB for 16 h, cell proliferation in all treatment groups significantly increased with the concentration of 3-HIB, with the 0.8 mM-1.6 mM treatment groups showing significantly higher cell proliferation than the low-concentration treatment groups (*p* < 0.05). Treatment with 3-HIB for 24 h did not affect cell proliferation. When cells were treated for 48 h, the proliferation results were similar to those at 16 h, with the 0.4–1.6 mM treatment groups showing significantly higher cell proliferation than the 0–0.2 mM concentration treatment groups (*p* < 0.05). The results of the EdU detection method were consistent with those of CCK8 method; the group treated with 0.8 mM 3-HIB demonstrated higher cell proliferation (Figure 1).

**Table 1:** Effect of 3-HIB supplementation on the proliferation of cells.

Groups	Treatment duration (h)					
	2	4	8	16	24	48
Con	0.48 ± 0.03	0.52 ± 0.02	0.53 ± 0.01	0.56 ± 0.02^c^	0.60 ± 0.02	0.47 ± 0.03^c^
0.2	0.51 ± 0.03	0.52 ± 0.01	0.54 ± 0.01	0.60 ± 0.02^b^	0.61 ± 0.01	0.52 ± 0.02^bc^
0.4	0.53 ± 0.01	0.55 ± 0.02	0.57 ± 0.02	0.63 ± 0.01^b^	0.62 ± 0.01	0.58 ± 0.02^a^
0.8	0.53 ± 0.03	0.58 ± 0.03	0.57 ± 0.03	0.71 ± 0.01^a^	0.63 ± 0.03	0.56 ± 0.02^ab^
1.6	0.47 ± 0.02	0.57 ± 0.02	0.57 ± 0.03	0.70 ± 0.01^a^	0.63 ± 0.04	0.60 ± 0.04^a^
SEM	0.01	0.01	0.01	0.01	0.02	0.02
*p*-value	0.390	0.184	0.413	0.001	0.083	0.011

Data are shown as mean ± SEM. Means not sharing the same letter are different (*P* < 0.05).

### Effects of 3-HIB supplementation on the expression of cell proliferation-related proteins

**Figure 2:**
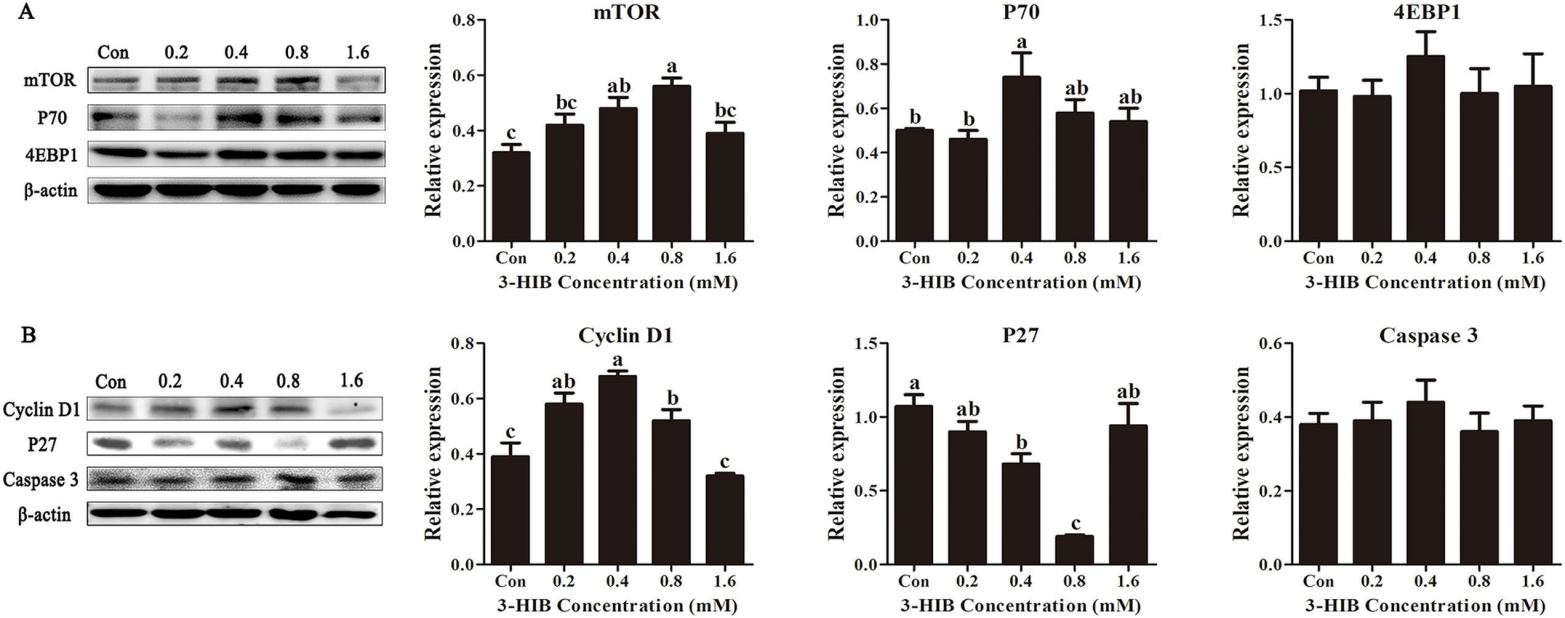
Effect of 3-HIB on the expression of cell proliferation-related proteins. **(A)** Detection of proteins related to the mTOR signaling pathway using Western blotting. **(B)** Detection of proteins related to cell proliferation and apoptosis using Western blotting. Data are shown as mean ± standard error of mean; means not sharing the same letter are significantly different (*p* < 0.05).

To investigate the mechanism of cell proliferation induced by 3-HIB, the expression levels of proteins related to cell proliferation and apoptosis were examined. Compared to the control group with no addition, the groups treated with 0.4 mM or 0.8 mM 3-HIB significantly increased the expression levels of cellular mTOR and its downstream P70 protein (*p* < 0.05), whereas the addition of 3-HIB did not affect the expression of 4EBP1 protein (*P* > 0.05) (Figure 2). The cell cycle-related protein expression tests showed that, consistent with the mTOR protein expression levels, the addition of 0.8 mM 3-HIB significantly increased the expression of Cyclin D1 protein while simultaneously inhibiting the expression of the cell cycle inhibitor protein P27 (*p* < 0.05). The addition of different concentrations of 3-HIB did not affect the expression of the apoptotic protein caspase-3.

### Effects of 3-HIB supplementation on the triglyceride and lipid droplet synthesis

**Figure 3:**
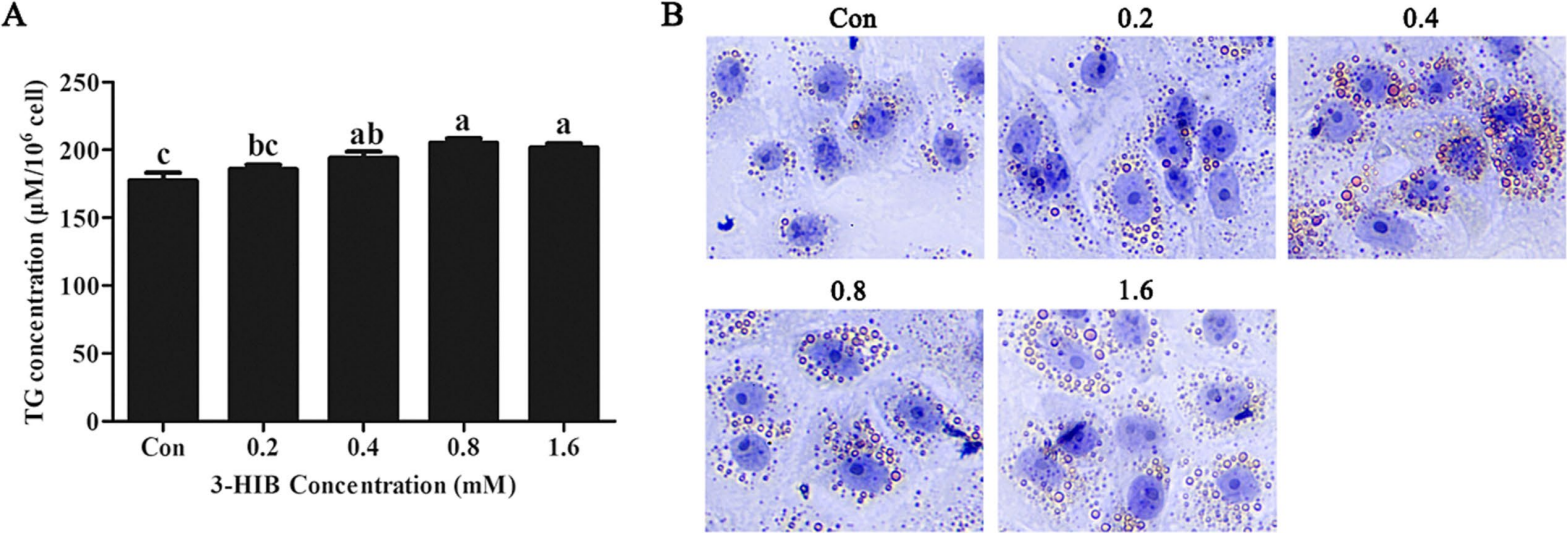
Effects of 3-HIB on cellular triglyceride and lipid droplet synthesis. **(A)** Detection of triglyceride synthesis using the Applygen Technologies, Inc. triglyceride test kit. **(B)** Detection of lipid droplet synthesis using Oil Red O staining. Data are shown as mean ± standard error of mean; means not sharing the same letter are different (*p* < 0.05).

Fatty acids are a primary source of cellular energy and we verified whether lipid metabolism affected cell proliferation. The addition of 0.4 mM or 0.8 mM 3-HIB to the culture medium significantly increased the concentration of cellular triglycerides compared to the control group (*p* < 0.05, Figure 3A). Oil Red O staining was used to observe cellular lipid droplet synthesis. Consistent with the triglyceride results, the synthesis of lipid droplets in the cells gradually increased with the addition of higher amounts of 3-HIB (Figure 3B).

### Effects of 3-HIB supplementation on the cellular fatty acid composition

To investigate why 3-HIB supplementation promoted the synthesis of cellular triglycerides and lipid droplets, cells were treated with an optimal dosage of 3-HIB (0.8 mM) and the control group (0 mM) for cellular fatty acid composition analysis. The addition of 3-HIB to the culture medium increased the content of various fatty acids in the cells (Table 2). The concentrations of long-chain fatty acids, such as C15:0, C20:0, and C22:0, in the cells were significantly higher than those in the control group (*p* < 0.05). Additionally, the concentrations of unsaturated fatty acids such as C16:1, C18:2, C20:3, and C20:4 in the cells were higher in the 3-HIB-supplemented group compared than in the control group (*p* < 0.05). This suggests that 3-HIB plays a functional role in promoting fatty acid transport in the cells.

**Table 2:** Effect of 3-HIB supplementation on the fatty acid composition of cells.

Fatty acids μg/10^7^ cells	Con	3-HIB	SEM	*p*-value
C14:0	1.08 ± 0.04	1.25 ± 0.05	0.05	0.054
C15:0	0.16 ± 0.01	0.19 ± 0.01	0.01	0.043
C16:0	19.89 ± 0.69	23.34 ± 1.04	0.95	0.050
C16:1	4.04 ± 0.15	4.73 ± 0.18	0.19	0.040
C18:0	16.84 ± 0.56	19.88 ± 0.98	0.85	0.054
C18:1 n-9	48.21 ± 1.72	55.40 ± 2.56	2.12	0.080
C18:2 n-6	1.89 ± 0.08	2.26 ± 0.10	0.10	0.046
C20:0	1.08 ± 0.04	1.32 ± 0.08	0.07	0.047
C20:1	5.36 ± 0.19	6.26 ± 0.35	0.27	0.087
C20:2	0.68 ± 0.02	0.83 ± 0.04	0.04	0.042
C20:3 n-6	2.37 ± 0.10	2.80 ± 0.12	0.12	0.049
C20:4 n-6	8.07 ± 0.28	9.52 ± 0.36	0.38	0.034
C22:0	0.57 ± 0.02	0.69 ± 0.04	0.03	0.044
C20:5 n-3	0.27 ± 0.01	0.32 ± 0.01	0.01	0.047
C22:1 n-9	0.91 ± 0.04	1.06 ± 0.06	0.05	0.106
C24:0	0.98 ± 0.04	1.13 ± 0.06	0.05	0.109
C24:1	0.56 ± 0.03	0.70 ± 0.06	0.04	0.095
C22:6	5.90 ± 0.15	6.83 ± 0.17	0.23	0.016

### Effects of 3-HIB supplementation on the expression of lipid metabolism-related proteins

**Figure 4:**
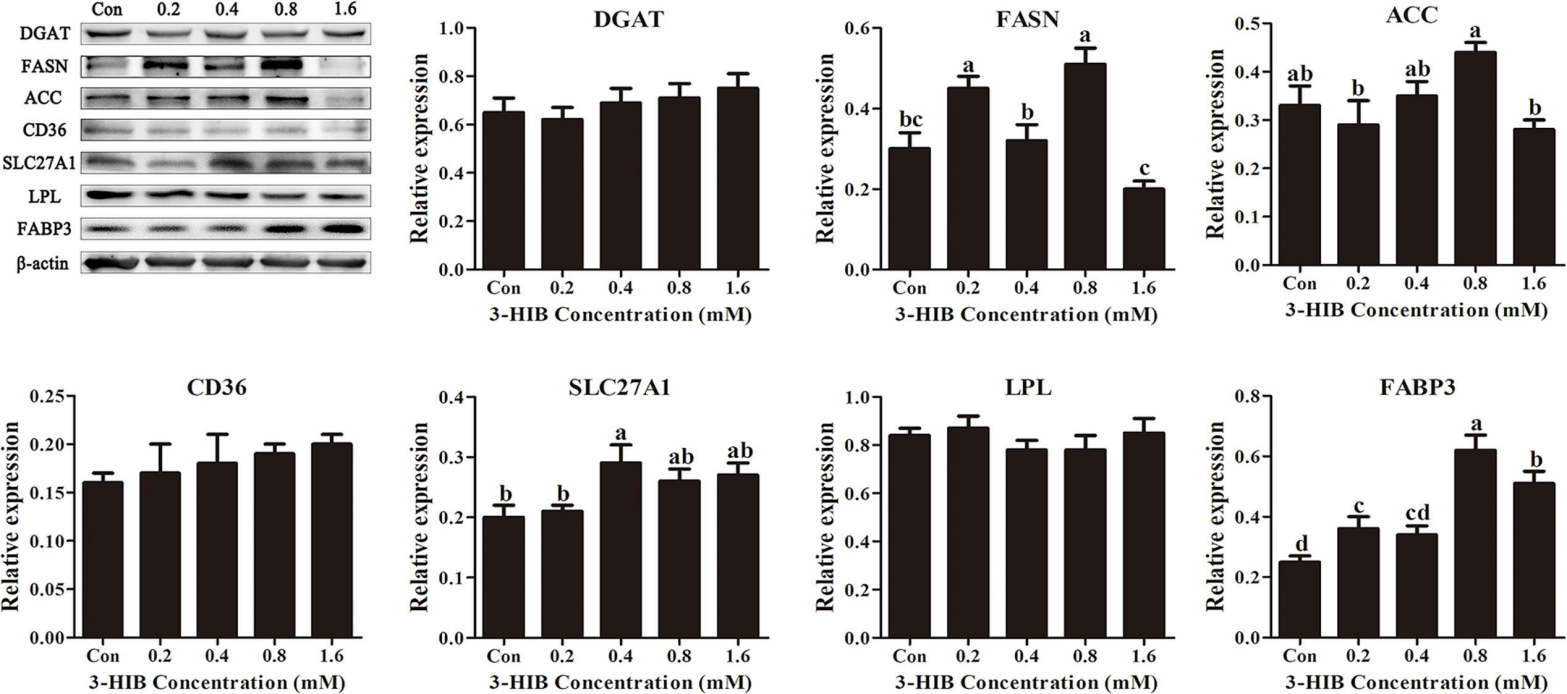
Effect of 3-HIB on the expression of lipid metabolism-related proteins. Data are shown as mean ± standard error of mean; means not sharing the same letter are different (*p* < 0.05).

To verify that the addition of 3-HIB promoted the transport of extracellular fatty acids into cells, the expression levels of proteins related to lipid metabolism, including those involved in fatty acid transport and *de novo* fatty acid synthesis were investigated. Regarding *de novo* fatty acid synthesis, the addition of 3-HIB did not affect the expression levels of the key proteins involved in this process, DGAT and ACC, relative to the control group (*p* > 0.05) (Figure 4). However, the expression levels of key proteins involved in cellular fatty acid transport, such as FABP3 and SLC27A1, were significantly increased in the group treated with 0.8 mM and 0.4 mM 3-HIB, respectively (*p* < 0.05). This demonstrates that 3-HIB promotes the transport of fatty acids in cells.

### Effects of 3-HIB supplementation on cellular ATP production

Further tests were conducted to determine whether the synthesized fatty acids increased oxidation to provide energy for cell proliferation. The effects of different concentrations of 3-HIB on ATP production were determined at 2, 4, 8, 16, 24, and 48 h (Table 3). The addition of different concentrations of 3-HIB to the culture medium did not affect cellular ATP production within the first 0–8 h. However, the addition of 0.8 mM or 1.6 mM 3-HIB to the culture medium significantly increased cellular ATP production compared to the control group at 16 and 24 h (*p* < 0.05). Addition of 3-HIB did not affect ATP production when the cells were cultured for 48 h (*p* > 0.05). This indicates that the addition of 3-HIB promotes the oxidation of cellular fatty acids to provide energy for cell proliferation.

**Table 3:** Effect of 3-HIB supplementation on the ATP production of cells.

Groups	Treatment duration (h)					
	2	4	8	16	24	48
Con	45.66 ± 3.31	52.61 ± 1.31	63.11 ± 3.94	74.00 ± 2.90^b^	67.81 ± 3.32^c^	66.33 ± 3.35
0.2	42.86 ± 3.21	53.61 ± 0.58	62.71 ± 2.05	73.83 ± 1.67^b^	76.44 ± 3.87^bc^	69.35 ± 2.35
0.4	40.97 ± 3.63	56.67 ± 2.12	62.66 ± 0.76	79.17 ± 2.07^b^	69.55 ± 4.43^c^	74.54 ± 3.37
0.8	44.46 ± 2.54	57.57 ± 2.97	69.72 ± 1.77	93.23 ± 3.48^a^	85.91 ± 3.42^ab^	74.50 ± 4.52
1.6	44.51 ± 2.87	57.26 ± 3.14	65.81 ± 2.22	93.49 ± 4.09^a^	88.57 ± 4.32^a^	74.00 ± 3.48
SEM	1.34	1.00	1.11	2.06	2.25	1.57
*P*-value	0.851	0.413	0.199	0.001	0.020	0.358

Data are shown as mean ± SEM. Means not sharing the same letter are different (*P* < 0.05).

### Effects of 3-HIB supplementation on the expression of fatty acid oxidation-related proteins

**Figure 5:**
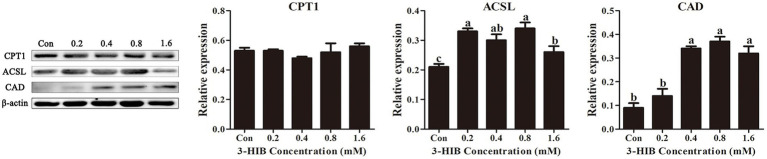
Effect of 3-HIB on the expression of fatty acid oxidation-related proteins. Data are shown as mean ± standard error of mean; means not sharing the same letter are different (*p* < 0.05).

To verify that the addition of 3-HIB increased fatty acid oxidation, the expression of proteins involved in fatty acid oxidation, including CPT-1, ACSL, and CAD, were determined (Figure 5). Adding 3-HIB to the culture medium does not affect the expression level of the key protein for fatty acid *β*-oxidation (CPT1) (*p* < 0.05). However, the expression levels of ACSL and CAD proteins were significantly elevated in the group treated with 0.8 mM 3-HIB compared to the control (*p* < 0.05). This indicates that an appropriate concentration of 3-HIB can regulate the expression of proteins related to fatty acid oxidation, promoting the *β*-oxidation process and ATP production.

## Discussion

Valine is a branched-chain amino acid that produces various intermediate products during metabolic processes in organisms (9). Of these intermediate products, 3-HIB has attracted widespread attention in recent years (18, 19). The current study found that 3-HIB could regulate the proliferation of PMECs by promoting fatty acid metabolism, including fatty acid transport and oxidative energy supply. This finding corresponds with the latest research progress (8), both domestically and internationally, and provides a new perspective for understanding the mechanism of action of valine or 3-HIB on cell proliferation.

Currently, research on 3-HIB regulation of lipid metabolism in cells has focused on humans and mice in human adipocytes (12) and mouse skeletal muscle cells (20), respectively. In human adipocytes, 3-HIB promotes the uptake of exogenous fatty acids by adipocytes, increases the substrate concentration for triglyceride synthesis, and inhibits 3-HIB synthase 3-hydroxyisobutyryl-CoA deacylase (HIBCH), substantially reducing lipid synthesis in cells (12). In mice, the intake of 3-HIB through drinking water or intramuscular injection leads to the accumulation of diacylglycerol in skeletal muscles, indicating that 3-HIB promotes lipid synthesis in mouse skeletal muscle cells (21). Knock-out of the HIBCH gene in mouse skeletal muscle cells inhibits the transport of fatty acids, whereas knock-out of the 3-HIB metabolic enzyme, 3-hydroxyisobutyryl-CoA dehydrogenase (HIBADH) to reduce the metabolic rate of 3-HIB, substantially increases cellular fatty acid intake (10). The studies indicate that 3-HIB can act as an agonist of fatty acid transport carriers, promoting the transport of more fatty acids into the cells for lipid synthesis, which is corroborated by the current study. To the best of our knowledge, this is the first time that 3-HIB has been shown to promote proliferation and fatty acid transport using PMECs.

In PMECs, fatty acids not only serve as essential nutrients to maintain normal cellular physiological functions but also become an important energy source for cell proliferation through *β*-oxidation (22). Fatty acids can act as signaling molecules that regulate key cellular signaling pathways (23). Abnormalities in fatty acid metabolism are often accompanied by cellular dysfunction (24). Recent studies have shown that 3-HIB affects fatty acid metabolism via various pathways, thereby regulating cell proliferation (8). In the current study, 3-HIB was found to promotes the expression of fatty acid transport proteins in PMECs, enhancing the ability of cells to take up fatty acids. The most abundant fatty acids in sow milk fat are long-chain fatty acids (LCFA), with palmitic and oleic acids being the most abundant, followed by linoleic acid, whereas the contents of medium-and short-chain fatty acids is very low (25). Medium-and short-chain fatty acids in mammary gland cells are synthesized *de novo* (26), whereas long-chain fatty acids mainly originate from the transportation of extracellular fatty acids into cells (27). Therefore, fatty acid transport plays a decisive role in milk fat synthesis. The current study found that 3-HIB increased the expression of fatty acid transport proteins, thereby enhancing the cellular uptake of fatty acids, increasing the content of long-chain fatty acids within the cells, and ultimately boosting the synthesis of triglycerides and lipid droplets. This finding is consistent with that of previous research on mice (21). Numerous studies have reported increased circulating valine and 3-HIB concentrations in obesity and insulin resistance, indicating that 3-HIB is closely related to cellular lipid metabolism (28, 29). The mechanism by which 3-HIB regulates cellular fatty acid transport remains unclear. Some studies have suggested that 3-HIB promotes the expression of fatty acid transport proteins by activating certain signaling pathways, such as the PI3K/Akt pathway (30).

Fatty acid oxidation is an important pathway for cellular energy production (31). In two stages, *β*-oxidation and *ω*-oxidation, fatty acids are converted to acetyl-CoA, which eventually enters the tricarboxylic acid cycle to produce energy (25). Previous studies in mice have found that the concentration of 3-HIB in plasma has become an important marker of the extent of mitochondrial fatty acid β-oxidation in mouse livers (32). The results of the current study indicate that 3-HIB promotes the activity of enzymes related to fatty acid oxidation in mammary gland cells and enhances the oxidative energy supply of fatty acids, thereby promoting cell proliferation. The 3-HIB can promote the process of fatty acid *β*-oxidation by upregulating the expression of proteins related to fatty acid oxidation, such as ACSL and CAD. This is consistent with the results of ATP synthesis in cells. However, there are no reports on the mechanism by which 3-HIB regulates cellular fatty acid oxidation. Some studies have shown that, in terms of fatty acid oxidation, 3-HIB may affect cellular metabolism through various pathways. The 3-HIB promotes mitochondrial respiratory chain activity, thereby increasing the efficiency of ATP synthesis within cells (32). In addition to directly promoting fatty acid metabolism, 3-HIB also indirectly affects the proliferation of PMECs through other pathways. For example, 3-HIB induces the expression of autophagy-related genes and promotes autophagy (33). Autophagy is the process of intracellular degradation and recycling of cellular components, and is important for maintaining cellular homeostasis and adapting to environmental changes (34). Studies have shown that 3-HIB promotes autophagy by upregulating the expression of autophagy-related genes (35), thereby providing the energy and materials necessary for cell proliferation. Therefore, further exploration of how 3-HIB affects the function of PMECs through autophagy is a worthwhile direction for future research.

## Conclusion

The study demonstrated that an appropriate concentration of 3-HIB can promote the proliferation of PMECs. This mechanism involves the ability of 3-HIB to regulate the expression of proteins related to fatty acid metabolism, thereby promoting the transport of fatty acids into the cells and enhancing intracellular fatty acid oxidation to produce ATP, which provides energy for cell proliferation. This study elucidates a new function of 3-HIB, and offers a novel explanation of the mechanism by which valine promotes the proliferation of PMECs.

## Data Availability

The original contributions presented in the study are included in the article, further inquiries can be directed to the corresponding author.
